# The Effect of Normothermic Machine Perfusion on the Immune Profile of Donor Liver

**DOI:** 10.3389/fimmu.2022.788935

**Published:** 2022-06-02

**Authors:** Andy Chao Hsuan Lee, Arianna Edobor, Maria Lysandrou, Vikranth Mirle, Amir Sadek, Laura Johnston, Ryan Piech, Rebecca Rose, John Hart, Beth Amundsen, Martin Jendrisak, James Michael Millis, Jessica Donington, Maria Lucia Madariaga, Rolf N. Barth, Diego di Sabato, Kumaran Shanmugarajah, John Fung

**Affiliations:** ^1^Department of Surgery, University of Chicago, Chicago, IL, United States; ^2^Biological Sciences Division, University of Chicago, Chicago, IL, United States; ^3^Pritzker School of Medicine, University of Chicago, Chicago, IL, United States; ^4^Department of Pathology, University of Chicago, Chicago, IL, United States; ^5^Gift of Hope Tissue and Donor Network, Itasca, IL, United States; ^6^Section of Transplant Surgery, Department of Surgery, University of Chicago, Chicago, IL, United States; ^7^Section of Thoracic Surgery, Department of Surgery, University of Chicago, Chicago, IL, United States

**Keywords:** normothermic machine perfusion, liver transplantation, immune profile, flow cytometry, immunohistochemistry

## Abstract

**Background:**

Normothermic machine perfusion (NMP) allows viability assessment and potential resuscitation of donor livers prior to transplantation. The immunological effect of NMP on liver allografts is undetermined, with potential implications on allograft function, rejection outcomes and overall survival. In this study we define the changes in immune profile of human livers during NMP.

**Methods:**

Six human livers were placed on a NMP device. Tissue and perfusate samples were obtained during cold storage prior to perfusion and at 1, 3, and 6 hours of perfusion. Flow cytometry, immunohistochemistry, and bead-based immunoassays were used to measure leukocyte composition and cytokines in the perfusate and within the liver tissue. Mean values between baseline and time points were compared by Student’s t-test.

**Results:**

Within circulating perfusate, significantly increased frequencies of CD4 T cells, B cells and eosinophils were detectable by 1 hour of NMP and continued to increase at 6 hours of perfusion. On the other hand, NK cell frequency significantly decreased by 1 hour of NMP and remained decreased for the duration of perfusion. Within the liver tissue there was significantly increased B cell frequency but decreased neutrophils detectable at 6 hours of NMP. A transient decrease in intermediate monocyte frequency was detectable in liver tissue during the middle of the perfusion run. Overall, no significant differences were detectable in tissue resident T regulatory cells during NMP. Significantly increased levels of pro-inflammatory and anti-inflammatory cytokines were seen following initiation of NMP that continued to rise throughout duration of perfusion.

**Conclusions:**

Time-dependent dynamic changes are seen in individual leukocyte cell-types within both perfusate and tissue compartments of donor livers during NMP. This suggests a potential role of NMP in altering the immunogenicity of donor livers prior to transplant. These data also provide insights for future work to recondition the intrinsic immune profile of donor livers during NMP prior to transplantation.

## Introduction

Liver normothermic machine perfusion (NMP) systems have gained clinical approval following recent clinical trials and are now in wider use (1). These devices may decrease the critical organ shortage by allowing the assessment and use of livers from extended criteria donors (2–7).

Liver NMP may also serve as a platform to condition organs prior to transplantation to minimize rejection or optimize organ function (8, 9). The liver contains high numbers of lymphohematopoietic cells that are transferred with donor livers to recipients during transplantation (10, 11). These donor leukocytes persist in the allograft following transplantation and have been shown to be important determinant of outcome (12). Using cell therapy or pharmacological treatment of allografts during NMP, the intrinsic immune function of donor livers could be manipulated to optimize organ survival, minimize primary graft dysfunction and offset rejection (8). Previous studies have demonstrated the favorable intrinsic immune properties of livers (13). In contrast with other solid-organ allografts, 8-33% of clinical liver transplant recipients who withdraw from immunosuppression exhibit operational tolerance, with an even higher incidence reported among pediatric patients (13, 14). The window of opportunity provided by NMP to recondition the immune profile of transplant livers therefore represents an important and clinically translatable research area.

It has been suggested that liver NMP may provide a less inflammatory environment than conventional cold storage, as NMP reduces the number of INF-gamma and IL-17-producing T cells and enlarges the regulatory T cell in the liver perfusates collected after NMP when compared to those collected after cold storage (15). In addition, studies using normothermic *ex vivo* lung perfusion demonstrated removal of passenger non-classical monocytes from perfusates, suggesting altered immunogenicity of transplant lungs with *ex vivo* perfusion (16, 17). These studies have analyzed the cellular and cytokine profile of circulating perfusate during NMP. Importantly the effect of NMP on the tissue-resident immune compartment that is transferred within the allograft has not been defined. In this study we determined serial changes in donor-liver resident leukocyte populations during NMP. In addition, we serially determined the leukocyte characteristics and cytokine profile within NMP perfusate. The results of this study reveal the effect of NMP on the immune profile of donor-livers and provide important insights into future strategies to manipulate the immune properties of livers during *ex vivo* perfusion.

## Methods

### Human Donor Liver Procurement

The study was reviewed and approved by the University of Chicago IRB under Request for Research on Decedents. The use of expired human donors’ organs for this research study was approved by Gift of Hope Tissue and Organ Network, Itasca, Illinois. Six non-transplantable human donor livers available for research were included in our study. Families of all human liver donors had consented donation of livers for research. Four donors were donation after cardiac death (DCD) and two were donation after brain death donors (DBD). Donors with history of hepatitis B or C infection and DCD donors who took longer than 90 minutes to expire after withdrawal of care were excluded from our study (**Table 1**). All liver grafts were procured by certified abdominal transplant surgeons registered as procuring surgeons by Gift of Hope Tissue and Organ Network, Itasca, Illinois, in standard clinical fashion. The liver grafts were then transported to our laboratory in standard cold storage fashion.

**Table 1:** Liver donor medical history and evaluation.

ID	Donor Age	Donor Gender	BMI	Donation Type	DCD time to expire (min)	Cold Ischemia Time (min)	Donor PMH	Reason for no transplant
6	73	Female	49.4	DBD	NA	291	Hypertension, 40 pack year smoker, HLD, OSA, MI, Schizophrenia, rhabdomyolysis	Cirrhosis on gross examination
7	57	Male	30.2	DCD	61	720	Hypertension, hyperlipidemia, obesity	Expedited DCD, not enough time to place liver
8	54	Male	19.9	DCD	67	664	Heart failure, cardiomyopathy, bradycardia, hyperlipidemia, COPD/emphysema, drug use, GERD, depression, CVA, >20 pack year smoker	DCD, prolonged time to expire
9	45	Male	23.9	DCD	12	407	ETOH abuse (1/5 vodka/day), COPD, HTN, Anxiety, >20 pack year smoker, recent cellulitis of left hand	70% macrosteatosis
10	49	Male	42.5	DBD	NA	422	Unknown	40% macrosteatosis
11	39	Male	42.1	DCD	9	393	25 pack year smoker, asthma, polysubstance abuse, obesity, depression	40% macrosteatosis

### Normothermic Machine Perfusion

**Figure 1:**
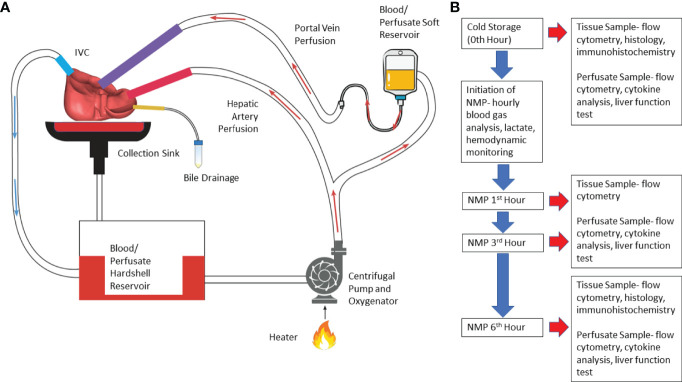
Illustration of normothermic machine perfusion set up and experimental design. **(A)** Schematic illustration- Perfusion of the circuit is propelled by a centrifugal pump; the perfusate passes through the heater and oxygenator where the perfusate is oxygenated by sweep gas that contains high concentration of O_2_ and low concentration of CO_2_; effluent perfusate from hepatic vein drains directly into the cannula which routes the perfusate to venous reservoir. **(B)** Experimental Design- Timeline of perfusion and sample collection. (NMP, normothermic machine perfusion).

Normothermic machine perfusion (NMP) of liver was performed using a circuit design compatible with existing clinical NMP devices (**Figure 1**). The circuit consisted of Sarns disposable centrifugal pump (Terumo, Ann Arbor, MI), CAPIOX FX Advance oxygenator with integrated hardshell venous reservoir (Terumo, Ann Arbor, MI), a soft venous reservoir bag (Terumo, Ann Arbor, MI), a collection basin coated with PTFE, and a combination of ¼ inch and 3/8-inch ECMO cannulas (Medtronic, Minneapolis, MN) (**Figure 1**). To simulate physiological pressures and flow rates, the portal vein was perfused by gravity *via* a soft venous reservoir bag that was hung 20 centimeters above the portal vein. The hepatic artery was cannulated and received inflow directly from the centrifugal pump. The inferior vena cava was cannulated from the superior cut end, and directed into the hardshell venous reservoir. The hardshell reservoir directed perfusate to the centrifugal pump, oxygenator and heater. Perfusate was subsequently divided between the hepatic artery and the soft venous reservoir bag prior to the portal vein. The common bile duct was cannulated separately with a pigtail cannula. Circuit perfusate consisted of 3-4 units of packed red blood cells obtained from the University of Chicago blood bank. Perfusate fluid was supplemented with 3 liters of DME-H21 high glucose (4500 mg/L) medium (Thermo Fisher Scientific, Waltham, MA) containing 5% bovine serum albumin, with an eventual hematocrit goal of 10-15%.

Perfusion was initiated at room temperature. Perfusate temperature was incrementally increased to a goal temperature of 36.8-37°C over 20 minutes. Sweep gas composed of 5L-7L/min O2 and 0.5-1L/min of CO_2_ was initiated to oxygenate inflow perfusate to the liver at the same time providing a physiologic level of dissolved CO_2_. Further titration of sweep gas was performed based on serial blood gas analysis. The centrifugal pump flow rate, or the total perfusate flow rate, was adjusted to achieve a desired ratio of portal vein flow rate to hepatic artery flow rate of 1:2 to 1:3. Six hours of perfusion was selected based on a clinically applicable time for machine perfusion. In addition, previous clinical and pre-clinical studies assessed donor organ viability during NMP when organs were perfused for an average of 4 hours (3–7).

### Physiological Measurements During NMP

Physiological parameters of the perfused liver were measured during NMP. The portal vein and hepatic artery pressures and flow rates were measured hourly. Three mL perfusate samples were collected hourly at both upstream and downstream of the organ for blood gas analysis to assess oxygenation, lactate and acid base status using an iSTAT point-of-care blood gas analyzer (Abbott Labs, Chicago, IL). Another 10 mL perfusate sample was collected hourly downstream of the organ for complete blood count and comprehensive metabolic panel including liver enzyme levels.

### Sample Processing

Before initiation of perfusion, perfusate samples from retrograde flush with cold phosphate-buffered saline (about 300 mL) and tissue samples from wedge biopsies (about 3 g) were obtained. The samples collected prior to initiation of perfusion were labeled as 0^th^ hour. During perfusion, perfusate samples (about 300 mL) and tissue samples (about 3 g) were obtained at each time point (1^st^ hour, 3^rd^ hour, and 6^th^ hour of perfusion). Perfusate samples were spun down at 400G and treated with ammonium-chloride-potassium buffer (Gibco|Thermo Fisher Scientific, Waltham, MA) to lyse remaining red blood cells. Liver tissue was mechanically disrupted and enzymatically digested at 37°C for 90 minutes using 100U/mL of Type II collagenase (Gibco|Thermo Fisher Scientific, Waltham MA) in Hank’s Balanced Salt Solution (Gibco|Thermo Fisher Scientific, Waltham MA) prior to filtration through 100-micron filters (Fisher Scientific, Waltham, MA). Samples were then washed and resuspended in phosphate-buffered saline.

### Flow Cytometry

Flow cytometry was used to determine the donor leukocyte profile in both the graft and perfusate compartments. Isolated cells were washed and stained with Zombie UV viability dye (Biolegend, San Diego, CA) in phosphate-buffered saline and then stained with the following markers: anti-CD3 Alexa Fluor 700 (Biolegend), anti-CD4 Alexa Fluor 488 (Biolegend), anti-CD8 Brilliant Violet 785 (Biolegend), anti-CD14 Brilliant Violet 650 (Biolegend), anti-CD16 Brilliant Violet 570 (Biolegend), anti-CD19 Brilliant Violet 510 (Biolegend), anti-CD45 Alexa Fluor 532 (Invitrogen, Carlsbad, CA), anti-CD56 PE-Vio770 (Miltenyl Biotec, Bergisch Gladbach, Germany), and anti-CD66b PE-dazzle (Biolegend) (**Supplemental Table 1**). Cells were identified and analyzed using an Aurora full spectrum flow cytometer (Cytek Biosciences, Fremont, CA), which offered the computational capability to subtract tissue autofluorescence from the actual stained samples. Analysis of flow cytometry results was performed using FlowJo Software (Version 10; FlowJo LLC, Ashland, OR) (**Supplemental Figure 1**).

### Histology and Immunohistochemistry

About 300 mg of tissue samples were taken as wedge biopsy prior to perfusion (0^th^ hour) and at the end of perfusion (6^th^ hour). Tissue samples were fixed in 10% neutral buffered formalin (Scigen, Gardena, CA) for 24-36 hours prior to embedding in paraffin. Sections were stained with hematoxylin and eosin for qualitative evaluation for sinusoidal and portal triad structural integrity.

Sections were also stained with anti-CD3 (BioCare medical, Pacheco, CA), CD4 (BioCare Medical), DAPI (Akoya Biosciences, Marlborough, MA) and FOXP3 (BioCare medical) (**Supplemental Table 2**), and slides were scanned by Vectra Polaris System (Akoya Biosciences) and analyzed with Inform Automated Image Analysis Software (Akoya Biosciences). A minimum of five regions of interest (ROI) (931 um x 698 um) were selected within each slide, in which number of tissue regulatory T cells (Treg cells) and CD3+ T cells were counted. The percentage of Treg cells out of all CD3+ T cells was calculated for each ROI.

### Cytokine Analysis

3mL of perfusate downstream from the organ were collected in ethylenediaminetetraacetic acid coated tube prior to perfusion and at 1^st^ hour, 3^rd^ hour, and 6^th^ hour of perfusion. Samples were spun down at 1200 RPM to obtain supernatant, which were immediately frozen on dry ice. The samples were then processed using Fisher Scientific Multiplex Assay, and the various cytokine levels were measured using Luminex 200 instrument system (Fisher Scientific).

### Statistical Analysis

Using flow cytometry, the frequency each immune cell type based on the total number of CD45+ cells was calculated in both perfusate and liver tissue. To illustrate the time dependent, dynamic effect of NMP on each individual cell type in either compartment, percentage change of each cell type frequency at various time points (1^st^ hour, 3^rd^ hour, or 6^th^ hour) relative to 0^th^ hour (cold storage) was calculated.


$$\text{Percentage Change at Time Point}\left(\text{x}\right)=100\%\times \frac{\text{cell frequency at time point }\left(\text{x}\right)-cell frequency at 0th hour}{cell frequency at 0th hour}$$

Paired student’s t-test was used to determine whether the percentage changes for each immune cell type at various time points referenced to 0^th^ hour were statistically significant. All values were presented as mean ± standard deviation. A P value < 0.05 was used for statistical significance.

Mean and standard deviation of concentration (pg/mL) of each cytokine at each time were calculated for all organs. Student’s t-test was used to determine if concentration changes between time points were significant.

Using immunohistochemistry, the percentage of regulatory T cells out of total number of CD3+ T cells were calculated for all ROI’s from each tissue slide taken from 0^th^ hour and 6^th^ hour. A mean regulatory T cell frequency was assigned to each organ for each time point by averaging all ROI values. Paired Student’s t-test was used to compare regulatory T cell frequencies of all organs between time points 0^th^ hour and 6^th^ hour.

## Results

### Normothermic Machine Perfusion Maintains Donor-Livers Under Physiological Conditions

**Figure 2:**
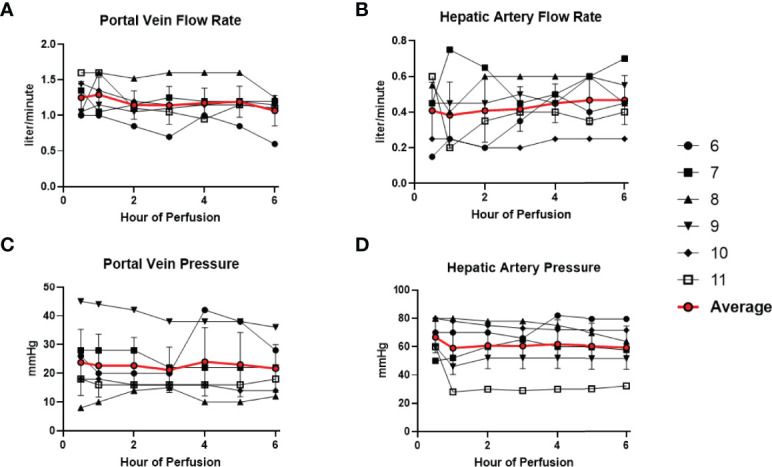
Normal physiologic parameters in all 6 livers were maintained during normothermic machine perfusion. **(A)** Portal vein flow rate (liter/minute) remained stable. **(B)** Hepatic artery flow rate (liter/minute) remained stable. **(C)** Portal vein pressure (mmHg) remained stable. **(D)** Hepatic artery pressure (mmHg) remained stable.

**Figure 3:**
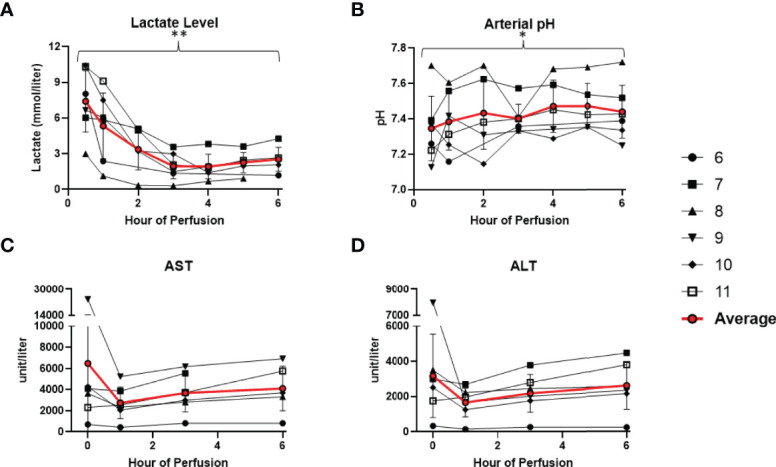
Metabolic parameters improved or remained stable in all 6 livers during normothermic machine perfusion. **(A)** Lactate level (mmol/liter) decreased during perfusion. **(B)** Hepatic artery pH normalized during perfusion. **(C)** Aspartate aminotransferase level (unit/Liter) remained stable. **(D)** Alanine aminotransferase level (unit/Liter) remained stable. (* P<0.05; ** P<0.01).

**Figure 4:**
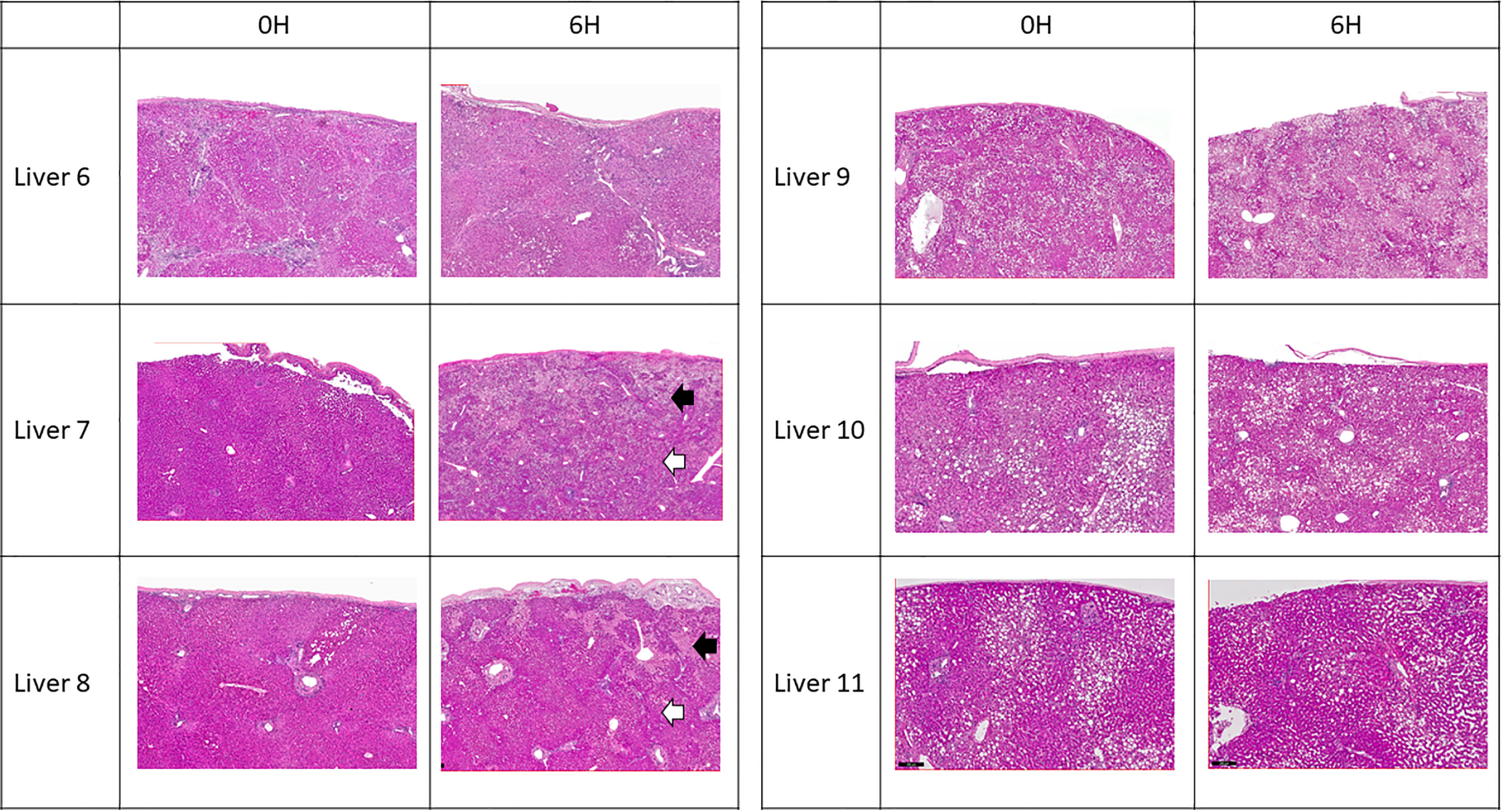
Representative H & E histology of liver tissue. Liver 6 exhibited cirrhotic change. Livers 9, 10 and 11 exhibited variable degree of microsteatotic change. At the end of perfusion, histology from livers 6, 9, 10, and 11 showed completely viable tissue with no structural change. Livers 7 and 8 exhibited patchy subcapsular hepatocyte necrosis (black arrows) with viable liver parenchyma underneath (white arrows).

All 6 donor-livers included in the study were successfully maintained on normothermic machine perfusion. Cold ischemia times varied from 291 to 720 minutes. Perfusion pressures and flow rates were constant and within physiologic range (**Figures 2A–D**). Lactate concentration fell to <3 by 6 hours in all livers except one (0.5^th^ h: 7.41 ± 2.58 versus 6^th^ h: 2.53 ± 1.01, *P* = 0.009) (**Figure 3A**). Following initiation of normothermic machine perfusion, acidosis within the circulating perfusate improved (0.5^th^ h: 7.35 ± 0.18 versus 6^th^ h: 7.44 ± 0.15, *P* = 0.047) (**Figure 3B**). Perfusate baseline lactate concentration varied. Aspartate aminotransferase level or alanine aminotransferase level within the perfusate stayed relatively constant throughout the duration of NMP (**Figures 3C, D**). Histological examination of liver tissue after 6 hours of perfusion showed structural integrity of underlying liver tissues with intact portal triad and sinusoids in all 6 livers. In the 2 DCD livers included in this study with longer than 60 minutes of warm ischemia time, focal superficial subcapsular hepatic necrosis was seen on histology. This was absent in the other four livers (**Figure 4**).

### Cellular Composition of Perfusate and Liver Tissue Is Altered During NMP

**Figure 5:**
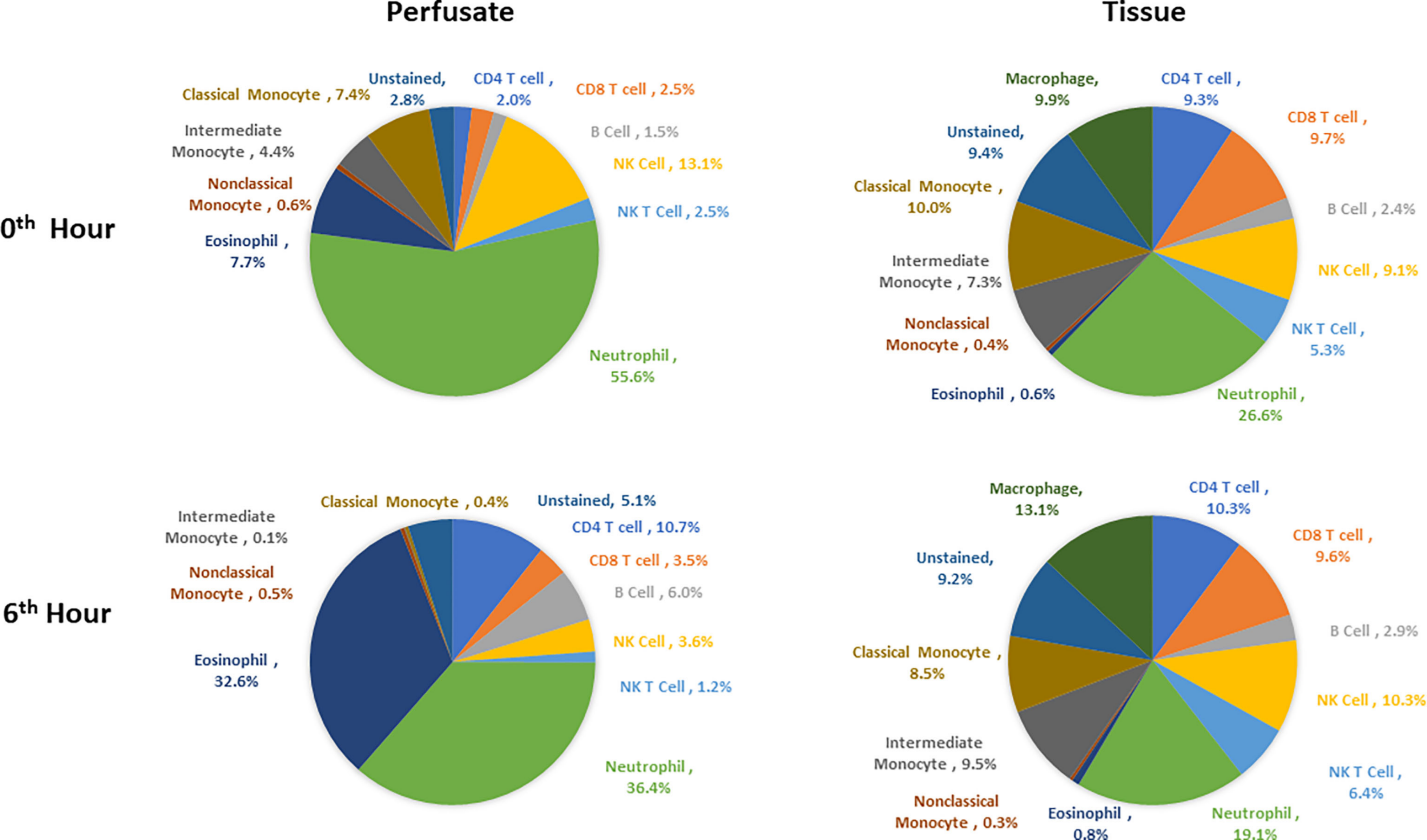
Composition of leukocytes of perfusates and liver tissues prior to perfusion and at the end of perfusion (N=6). Neutrophils were the most abundant cell types in both perfusates and tissues both prior to and at 6^th^ hour of perfusion. Percentages were obtained by dividing the count of each immune cell type by CD45+ cell count for each tissue type at each time point.

Following organ procurement and cold flush of donor-livers, the most abundant resident leukocytes in digested liver tissue were neutrophils (26.6 ± 19.7%), classical monocytes (10.0 ± 4.2%), and macrophages (9.9 ± 7.3%). By contrast, the composition of leukocytes within the perfusate consisted of predominantly neutrophils (55.6 ± 16.3), NK cells (13.1 ± 6.5%), and eosinophils (7.7 ± 10.8%) (**Figure 5**).

Changes in leukocyte composition were seen following NMP. After 6 hours on NMP, resident neutrophils remained the most abundant leukocyte in the tissue, but there was a decrease in the frequency of neutrophils within the overall leukocyte compartment (19.1 ± 17.8%). The second and third most abundant cells types in the liver tissues were macrophages (13.1 ± 10.0%) and NK cells (10.3 ± 7.1%). Similar to analysis of the tissue resident leukocytes, neutrophils were the most abundant leukocytes in the perfusate following 6 hours of NMP, with a lower percentage of neutrophils detected at 6 hours compared to prior to initiation of NMP (36.4 ± 24.5%). The second most abundant cell type were eosinophils (32.6 ± 22.6%), while CD4 T cells constituted a greater relative population following NMP (10.7 ± 4.7%) (**Figure 5**).

### Temporal Changes in Tissue Resident Leukocyte Composition During Normothermic Machine Perfusion

Dynamic changes in immune cell composition are seen during the course of NMP. To serially determine the changes in tissue leukocyte composition during NMP, the percent change of each cell type was evaluated at various time points relative to prior to initiation of NMP.

**Figure 6:**
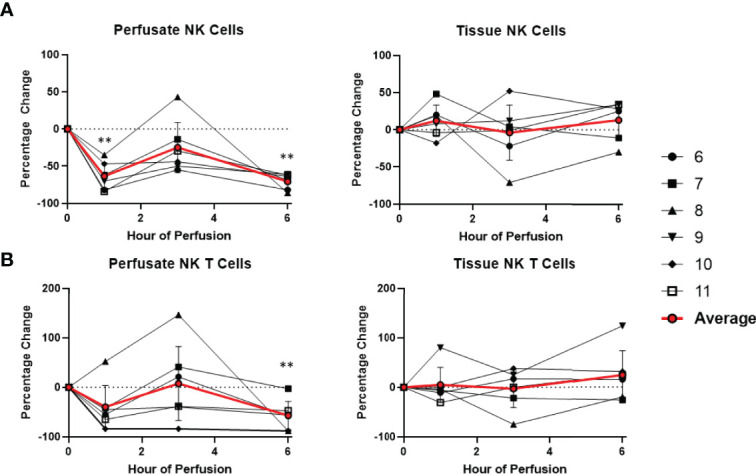
Percentage change of frequency of cells from NK cell lineage during perfusion. A percentage change of 0% suggests there is no change in the innate immune cell frequency from time point 0^th^ hour. Average values of percentage changes (red line) as well as standard deviations were calculated (vertical bar). A two-sided, paired t-test was performed to compare percentages of each time point to those at time 0H. (** P<0.01). **(A)** Perfusate NK cell frequency decreased at the 1^st^ hour of perfusion (-63.4 ± 17.8%) and remained decreased at the 6^th^ hour of perfusion (-70.7 ± 9.8%), without changes to tissue NK cell frequency. **(B)** Perfusate NK T cell frequency decreased at the 6^th^ hour of perfusion (-56.8 ± 28.5%) without changes to tissue NK T cell frequency.

**Figure 7:**
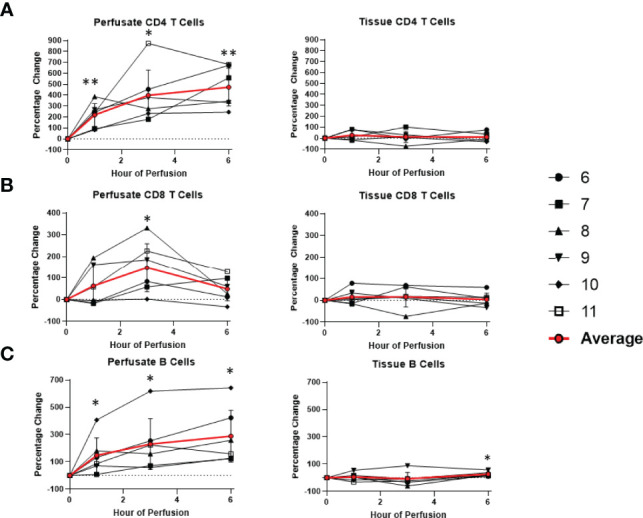
Percentage change of frequency of cells from lymphocyte lineage during perfusion. A percentage change of 0% suggests there is no change in the innate immune cell frequency from time point 0^th^ hour. Average values of percentage changes (red line) as well as standard deviations were calculated (vertical bar). A two-sided, paired t-test was performed to compare percentages of each time point to those at time 0H. (* P<0.05; ** P<0.01). **(A)** Perfusate CD4 T cell frequency increased at the 1^st^ hour of perfusion (+218.9 ± 104.6%) and continued to increase at the 6^th^ hour (+472.6 ± 172.5%) without changes to tissue CD4 T cell frequency. **(B)** Perfusate CD8 T cell frequency increased at the 3^rd^ hour of NMP (+147.4 ± 111.3%) and returned to baseline at the 6^th^ hour without changes to tissue CD8 T cell frequency. **(C)** Perfusate B cell frequency increased at the 1^st^ hour of perfusion (+147.4 ± 127.5%) and continued to increase at the 6^th^ hour of perfusion (+288.5 ± 189.2%), while tissue B cell frequency increased at 6^th^ hour of perfusion (+26.3 ± 16.0%).

Overall, there was no significant changes within the T cell and NK cell compartments within the tissue among tissue CD8 T cells, CD4 T cells, NK cells, and NK T cells. Tissue B cells constituted a significant higher proportion of leukocytes at the end of normothermic perfusion, with no significant differences observed at earlier time points (+26.3 ± 16.0%) (**Figures 6**, **7**).

**Figure 8:**
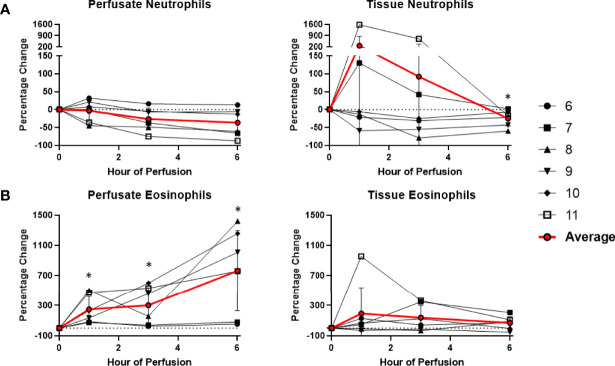
Percentage change of frequency of cells from granulocyte lineage during perfusion. A percentage change of 0% suggests there is no change in the innate immune cell frequency from time point 0^th^ hour. Average values of percentage changes (red line) as well as standard deviations were calculated (vertical bar). A two-sided, paired t-test was performed to compare percentages of each time point to those at time 0H. (* P<0.05). **(A)** No changes were observed in perfusate neutrophil frequency but tissue neutrophil frequency decreased at the 6^th^ hour of perfusion (-24.5 ± 21.3%). **(B)** Perfusate eosinophil frequency increased at the 1^st^ hour of perfusion (+249.3 ± 175.3%) and continued to increase at the 6^th^ hour of perfusion (+763.1 ± 533.4%), without changes to tissue eosinophil frequency.

**Figure 9:**
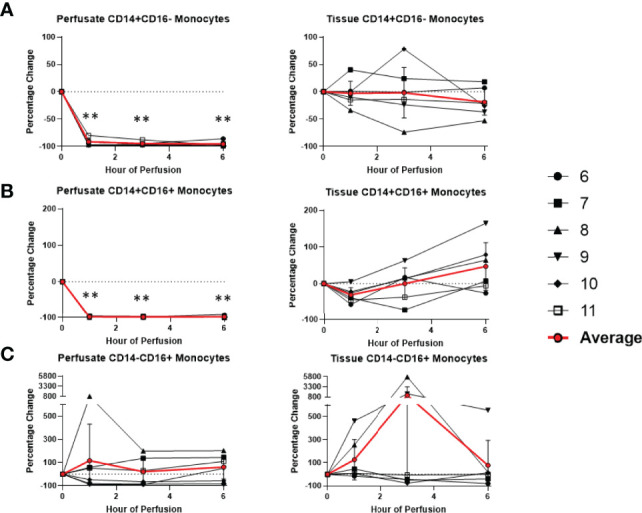
Percentage change of frequency of cells from monocyte lineage during perfusion. A percentage change of 0% suggests there is no change in the innate immune cell frequency from time point 0^th^ hour. Average values of percentage changes (red line) as well as standard deviations were calculated (vertical bar). A two-sided, paired t-test was performed to compare percentages of each time point to those at time 0H. (** P<0.01). **(A)** Perfusate classical monocyte frequency decreased at the 1^st^ hour of perfusion (-91.6 ± 5.8%) and remained decreased at the 6^th^ hour of perfusion (-95.6 ± 4.3%), with no changes observed in tissue classical monocyte frequency. **(B)** Perfusate intermediate monocyte frequency decreased at the 1^st^ hour of perfusion (-98.3 ± 1.5%) and remained decreased at the 6^th^ hour of perfusion (-98.5 ± 2.7%), while tissue intermediate monocyte frequency decreased at the 1^st^ hour of perfusion (-53.2 ± 17.6%) and returned to baseline at the 3^rd^ hour of perfusion. **(C)** No changes were observed in perfusate non-classical monocyte frequency and tissue non-classical monocyte frequency.

Among cells of myeloid lineage, the frequency of tissue neutrophils was significant lower at the 6^th^ hour of NMP (-24.5 ± 21.3%) (**Figure 8**). The frequency of tissue intermediate monocytes dropped in the tissue at the 1^st^ hour (-30.7 ± 19.7%). By the third hour of perfusion, the frequency of tissue intermediate monocyte was similar to baseline (**Figure 9**). No significant change existed in frequencies of tissue eosinophil, classical and non-classical monocytes during NMP compared to cold storage baseline (**Figures 8**, **9**).

### Temporal Changes in Perfusate Leukocyte Composition During Normothermic Machine Perfusion

Dynamic changes in the composition of perfusate leukocytes were seen during NMP. Among cells of lymphoid lineage, perfusate CD4 T cell frequency significantly increased at the 1^st^ hour of NMP (+218.9 ± 104.6%) and continued to increase at the 6^th^ hour (+472.6 ± 172.5%). Perfusate B cell frequency likewise increased throughout duration of NMP, starting at the 1^st^ hour (+147.4 ± 127.5%) and continuing to the 6^th^ hour (+288.5 ± 189.2%) (**Figure 7**). On the other hand, a significantly lower proportion of perfusate NK cell was observed (-63.4 ± 17.8%) at the 1^st^ hour of perfusion and remained decreased at the 6^th^ hour of perfusion (-70.7 ± 9.8%) (**Figure 6**). Similarly, perfusate NK T cell frequency decreased at the 6^th^ hour of NMP (-56.8 ± 28.5%) (**Figure 6**). Perfusate CD8 T cell frequency at the 6^th^ hour was comparable to that seen prior to initiation of NMP (**Figure 7**).

Among cells of myeloid lineage, perfusate eosinophil frequency increased throughout the duration of perfusion, from (+249.3 ± 175.3%) at the 1^st^ hour to (+763.1 ± 533.4%) at the 6^th^ hour (**Figure 8**). In contrast, perfusate classical monocytes and intermediate monocyte frequencies both immediately decreased following initiation of NMP at the 1^st^ hour (-91.6 ± 5.8% and -98.3 ± 1.5%, respectively), and remained decreased throughout NMP (**Figure 9**). No significant change was observed in perfusate neutrophil and non-classical monocyte frequencies throughout NMP (**Figures 8**, **9**).

### Effect of NMP on Tissue Resident T Regulatory Cells

**Figure 10:**
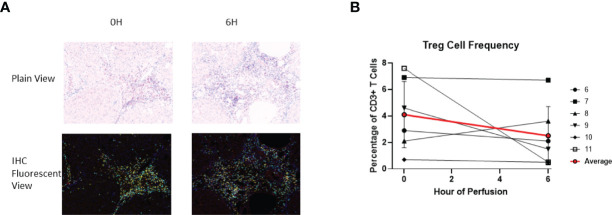
Identification of Treg using IHC (DAPI+CD3+CD4+FoxP3+). **(A)** Representative Slides (Liver 6) from before (0^th^ hour) and following perfusion (6^th^ hour). DAPI (Blue), CD3 (Cyan), CD4 (Yellow), FoxP3 (Red). **(B)** Treg cell frequency out of total CD3+ T cells both before and following perfusion.

Given that FoxP3, a critical marker for regulatory T cells (Treg cells), is a transcription factor, immunohistochemistry was used to identify tissue Treg cells. At baseline, 4.1 ± 2.5% of tissue CD3+ T cells were Treg cells. At the end of perfusion, 2.5 ± 2.2% of tissue CD3+ T cells were Treg cells. Compared to baseline, there was no significant T reg cell frequency change at end of perfusion (**Figure 10**). Within individual livers changes to Treg composition were observed, with increase in liver 8 and decrease in liver 9, 10 and 11.

### The Effect of NMP on Cytokine Secretion

Among pro-inflammatory cytokines, IL-4, IL-17A, IFN-gamma, and TNF-alpha were below the limit of detection in cold storage. At one hour after initiation of NMP, there was significant increase in IL-2, IL-4, and GM-CSF compared to cold storage. Compared to their levels at 1 hour, IL-1b, IL-2, IL-4, IL-6, G-CSF, IFN-gamma, GM-CSF, TNF-alpha, CXCL2, and CXCL1 continued to increase at 3^rd^ hour of NMP. By the 6^th^ hour of perfusion, compared to cold storage, there were significant increases in IL-4, IL-6, IL-8, G-CSF, GM-CSF, CCL2, CXCL2, CXCL1 and granzyme A. No significant differences were detected in levels of IL-1b, IL-2, IL-17A, IFN-gamma, TNF-alpha and granzyme B between cold storage and at 6th hour of perfusion, despite significant temporary increases in levels of IL-1b, IL-2 and TNF-alpha at the 3^rd^ hour of perfusion (**Table 2**).

**Table 2:** Cytokine concentrations (pg/mL) in human livers during 6 hour of normothermic machine perfusion.

	Hour 0 of NMPConcentration (pg/mL)	Hour 1 of NMPConcentration (pg/mL)	1H vs 0H P-value	Hour 3 of NMPConcentration (pg/mL)	3H vs 0H P-value	3H vs 1H P-value	Hour 6 of EVLPConcentration (pg/mL)	6H vs 0H P-value	6H vs 1H P-value
IL-1b	8.39 ± 11.08	1.92 ± 0.77	0.226	37.14 ± 26.06	0.086	0.029	252.16 ± 261.41	0.095	0.085
IL-2	1.47 ± 0.43	3.04 ± 1.28	0.023	13.54 ± 7.44	0.016	0.020	37.45 ± 45.91	0.140	0.150
IL-4	0.60 ± 0.80	5.21 ± 3.44	0.037	25.44 ± 3.13	0.000	0.000	24.27 ± 2.91	0.000	0.000
IL-6	14.10 ± 8.12	567.37 ± 628.36	0.106	26368.84 ± 17769.95	0.021	0.020	24707.60 ± 17412.95	0.025	0.025
IL-8 (CXCL8)	43.40 ± 73.04	2815.07 ± 3103.37	0.105	2835.61 ± 1563.92	0.012	0.989	785.97 ± 267.48	0.002	0.197
IL-10	0.19 ± 0.12	186.28 ± 128.71	0.023	3223.56 ± 2154.59	0.020	0.025	4686.07 ± 2901.50	0.015	0.016
IL-17A (CTLA-8)	0.09 ± 0.13	0.22 ± 0.03	0.194	4.00 ± 5.32	0.163	0.180	19.22 ± 21.92	0.108	0.110
G-CSF (CSF-3)	1.58 ± 2.38	0.94 ± 1.80	0.614	1088.88 ± 318.49	0.001	0.001	2482.11 ± 688.53	0.000	0.000
IFN-gamma	0.20 ± 0.12	0.21 ± 0.16	0.941	6.90 ± 5.30	0.038	0.035	220.75 ± 245.05	0.100	0.100
GM-CSF	2.04 ± 0.95	5.91 ± 2.99	0.025	27.11 ± 5.76	0.000	0.000	53.31 ± 35.74	0.023	0.030
TNF-alpha	0.03 ± 0.07	205.68 ± 233.06	0.104	2633.87 ± 2027.38	0.034	0.031	1488.60 ± 1331.19	0.054	0.056
MCP-1 (CCL2)	220.74 ± 117.06	916.22 ± 736.96	0.109	3200.63 ± 3030.23	0.075	0.169	1622.62 ± 706.26	0.006	0.136
MIP-2a (CXCL2)	4.56 ± 4.83	526.88 ± 589.60	0.105	2040.39 ± 1209.96	0.013	0.022	1930.59 ± 996.82	0.008	0.017
GROa (CXCL1)	1.28 ± 2.70	76.63 ± 65.93	0.054	377.16 ± 106.55	0.001	0.001	297.82 ± 69.17	0.000	0.000
Granzyme A	29.80 ± 30.54	61.97 ± 55.69	0.291	87.56 ± 54.01	0.012	0.310	114.26 ± 66.80	0.022	0.019
Granzyme B	5.04 ± 1.19	9.97 ± 4.53	0.078	9.54 ± 4.24	0.071	0.880	125.79 ± 139.88	0.112	0.127

The following cytokines were presented in abbreviations in the table: interleukin-1b (IL-b), interleukin-2 (IL-2), interleukin-4 (IL-4), interleukin-6 (IL-6), interleukin-8 (IL-8) or C-X-C motif chemokine ligand 8 (CXCL8), interleukin-10 (IL-10), interleukin 17-A (IL-17A) or cytotoxic T-lymphocyte-associated antigen 8 (CTLA-8), granulocyte colony-stimulating factor (G-CSF) or colony stimulating factor 3 (CSF-3), interferon-gamma (IFN-gamma), granulocyte-macrophage colony-stimulating factor (GM-CSF), tumor necrosis factor-alpha (TNF-alpha), monocyte chemotactic protein-1 (MCP-1) or C-X-C motif chemokine ligand 2 (CXCL-2), and growth-regulated oncogene alpha (GROa) or C-X-C motif chemokine ligand 1 (CXCL1). Data are presented as mean ± standard deviation.

With regards to anti-inflammatory cytokines, IL-10 was below the limit of detection in cold storage. However, IL-10 consistently increased throughout duration of NMP starting at the 1^st^ hour of perfusion. At the 6^th^ hour of perfusion, IL-10 had more than 20,000-fold increase compared to baseline cold storage (**Table 2**).

## Discussion

NMP devices allow *ex vivo* viability assessment and have been shown to allow successful transplantation of livers that would otherwise be discarded based on specific high-risk criteria (2, 4, 5). In this study we demonstrate serial changes in composition of passenger leukocytes of livers maintained on NMP. Within the donor-liver tissue, a significant increase in cell frequency of B cells and decrease in cell frequency of neutrophils were detected at the end of NMP with stable cell frequencies of other cell lineages. When serial perfusate was analyzed, duration of NMP was associated with increases in frequencies of CD4 T cell, B cells and eosinophils and significant decreases in frequencies of NK cells, classical monocytes and intermediate monocytes. Cytokine analysis demonstrated increased levels of cytokines associated with lymphocytes (IL-2, IL-4, IL-6), granulocytes (CXCL2, CXCL1, G-CSF, IL-8), NK cells (granzyme A, interferon-gamma), monocytes/macrophages (CCL-2, GM-CSF, interferon gamma), as well as increased levels of anti-inflammatory cytokine associated with lymphocytes and macrophages (IL-10) during NMP.

Previous ex vivo perfusion studies have demonstrated an effect of normothermic machine perfusion on the immune profile of organs. NMP reduced the number of INF-gamma and IL-17-producing T cells and enlarged the regulatory T cell in the liver perfusates collected after NMP compared to cold storage (15). In addition, lungs maintained on *ex vivo* lung perfusion (EVLP) demonstrate a rapid increase in perfusate monocytes at onset of perfusion with abundant non-classical monocytes attached to the leukocyte filter at end of EVLP, suggesting EVLP may remove passenger non-classical monocytes (16). In contrast to previous work assessing leukocyte populations in perfusate during or after machine perfusion of organs, we have characterized the immune profile of liver tissue during machine perfusion in liver. Given that the liver tissue rather than the perfusate is transferred and the immune cells within the graft has been shown to have important immunological effects, our results may have important implications on future work.

We have demonstrated that NMP significantly increased the proportion of T cells that are detectable in the perfusate throughout the course of perfusion. This may suggest that donor tissue T cells are mobilized into the perfusate during NMP. This finding may not be surprising, considering the migration of donor passenger T cells from the donor liver allograft into recipient circulation has been demonstrated in the literature prior to clinical use of NMP (10, 11). However, tissue T cell frequency stayed relatively unchanged throughout the course of NMP. There are several possible explanations for this including that the liver contains a large reservoir of T cells relative to the perfusate. As a result, the increase in detectable T cells within the perfusate during perfusion may represent a small amount of the liver resident T cells. An alternative explanation is that perfusate T cells continuously migrate back into the donor liver tissue, creating a dynamic T cell trafficking loop between the perfusate and tissue compartments. The increased levels of T helper type 1 cell cytokines (IL-2, IFN-gamma), T helper type 2 cell cytokines (IL-4, IL-6) and T cell-associated anti-inflammatory cytokine (IL-10) demonstrate that multiple counteracting molecular pathways are present and may orchestrate a balanced cell migration equilibrium.

We have demonstrated that tissue neutrophil frequency significantly decreased at end of normothermic perfusion, though no significant change was observed in the perfusate neutrophils. The drop in tissue neutrophil frequency suggests that tissue neutrophils are activated and mobilized during NMP. This observation be may explained by ischemia-reperfusion injury of the circuit. In our study, cold ischemia time of donor-livers ranged from 4 hours to 12 hours. The ischemic period was followed by reperfusion on NMP, where there was a shift from metabolic distress caused by ischemia to an excessive innate immune response triggered by reperfusion. The reperfusion phase may induce cell damage within the donor-livers and result in the release of endogenous molecules that served as chemoattractant to neutrophils, as observed by increasing levels of CXCL1 and CXCL2 in the perfusates during NMP (18). Of note, reperfusion of donor-livers in recipients is historically known to cause excessive neutrophil influx to the liver from the vasculature, causing injury following reperfusion (18). However, in NMP, such endogenous source of circulating recipient neutrophils is lacking, and may explain the paradoxical decrease in donor-liver tissue neutrophils that we observed. Interestingly, we did not observe increased perfusate neutrophil cell frequency during NMP, contrary to what we would expect. One potential explanation may be that the exposure of circulating neutrophils in perfusates to non-endothelialized surfaces of the perfusion circuit may cause proinflammatory state, resulting in adherence of neutrophils to circuits. Our findings are consistent with previous studies demonstrating reduced numbers of neutrophil clusters in liver tissue following NMP compared to cold storage with the decrease attributed to neutrophil circulation and possibly to an anti-inflammatory hepatic environment during NMP (15). Neutrophils have been recognized as a key player in liver injury following ischemia reperfusion, and elimination of excessive neutrophils or inhibition of their function may lead to reduction of liver injury and inflammation following transplantation (17). The effect of selective depletion of tissue neutrophils following NMP will be determined in future studies.

However, unlike previous studies, the current study initiated NMP after transport with associated cold ischemia time (4, 5, 15). Despite subjecting each donor liver to different length of cold ischemia time, we observed similar findings in all livers with increased frequencies of perfusate CD4 T cells, B cells and eosinophils and decreased frequency of tissue neutrophils during NMP. This can likely be explained by the controlled oxygenated rewarming employed by our study after substantial cold ischemia time. In prior animal studies, NMP with controlled oxygenated rewarming of liver over an extended period of time after cold storage was shown to result in significantly improved recovery and histopathology upon reperfusion as compared to cold-stored only livers (19, 20). This suggested beneficial role of NMP even after significant cold ischemia time, and the protective role of controlled oxygenated rewarming against liver injury following due to prolonged cold ischemia time.

There are limitations in our study. First of all, the livers included in our study did not meet transplant criteria prior to perfusion. Using these discarded livers, we were able to take serial wedge biopsies to characterize the organ-resident immune profile. While this study would not be possible in livers used to clinical transplant, the livers used are similar in profile to high-risk livers that have been investigated in clinical trials (VITTAL) (5). All livers included in our study showed ability to clear elevated levels of lactate and transaminases that accumulated during cold ischemia. The observed increased cytokine concentrations throughout perfusion suggested that decreased levels of lactate and transaminases during machine perfusion were not due to potential dilution alone, but may instead suggested preserved liver metabolism (21). The results of the study may therefore be applicable to the cohort of organs that will be placed in machine perfusion systems in the future for feasibility assessment. Future studies will determine whether changes to immune profile of organs influences liver survival during NMP. Secondly, we utilized stored pRBC’s that were discarded from clinical use or expired from local blood bank and permissively utilized perfusates with low hematocrits to preserve blood product usage. The maximal time that pRBC’s were used for clinical care was 42 days from original date of donation. With our perfusion system, all livers’ perfusion parameters were stable with viable liver parenchymal cells on histology. We acknowledge that expired pRBC’s may alter immunology. However, recent preclinical trials on NMP similarly utilized stored pRBC’s (22). Third of all, we chose to demonstrate our data with cell frequency rather than absolute amounts. This allows comparison between time points and is compatible with flow for detailed cell phenotype data. Cell frequency also allows better reproducibility of results across experiments (23). Finally, it is possible that perfusate passenger leukocytes were consumed by the perfusion circuit during NMP due to cell lysis or cell adhesion to the perfusion tubes, thus contributing to the trends we have observed. However, this phenomenon may be present in clinical NMP as well.

In summary, our study demonstrates changes to the intrinsic immune profile of liver donor-livers during NMP. Furthermore, the changes in immune profiles of the perfusate and the organ don’t seem to mirror each other. Given that the organ is transplanted while the perfusate is discarded, our findings may be important in future NMP studies to address this. The results of our study have important implications on future work on novel targeted therapies to recondition donor livers using NMP to expand the organ donor poor, prevent delayed graft failure and induce tolerance.

## Data Availability Statement

The original contributions presented in the study are included in the article/**Supplementary Material**. Further inquiries can be directed to the corresponding author.

## Author Contributions

AL - study design, data acquisition, data analysis, and writing of manuscript. AE - data acquisition and writing of manuscript. ML- data acquisition and writing of manuscript. VM - data acquisition and writing of manuscript. AS - data acquisition and writing of manuscript. LJ - data acquisition, data analysis, and writing of manuscript. RP - data acquisition. RR - data acquisition. JH - data acquisition. BA - data acquisition. MJ - data acquisition. JM - study design, supervision, and manuscript revision. JD - acquisition of funding and supervision. MM - acquisition of funding, study design, and supervision. RB - acquisition of funding and supervision. DS - acquisition of funding and supervision. KS - acquisition of funding, study design, supervision, and manuscript revision. JF - acquisition of funding, supervision, and manuscript revision. All authors contributed to the article and approved the submitted version.

## Conflict of Interest

The authors declare that the research was conducted in the absence of any commercial or financial relationships that could be construed as a potential conflict of interest.

## Publisher’s Note

All claims expressed in this article are solely those of the authors and do not necessarily represent those of their affiliated organizations, or those of the publisher, the editors and the reviewers. Any product that may be evaluated in this article, or claim that may be made by its manufacturer, is not guaranteed or endorsed by the publisher.
