# Supplementary figures and images for: The Effect of Normothermic Machine Perfusion on the Immune Profile of Donor Liver
Source: Front Immunol. 2022 Jun 2;13:788935. doi: 10.3389/fimmu.2022.788935 (PMC9201055; doi:10.3389/fimmu.2022.788935)

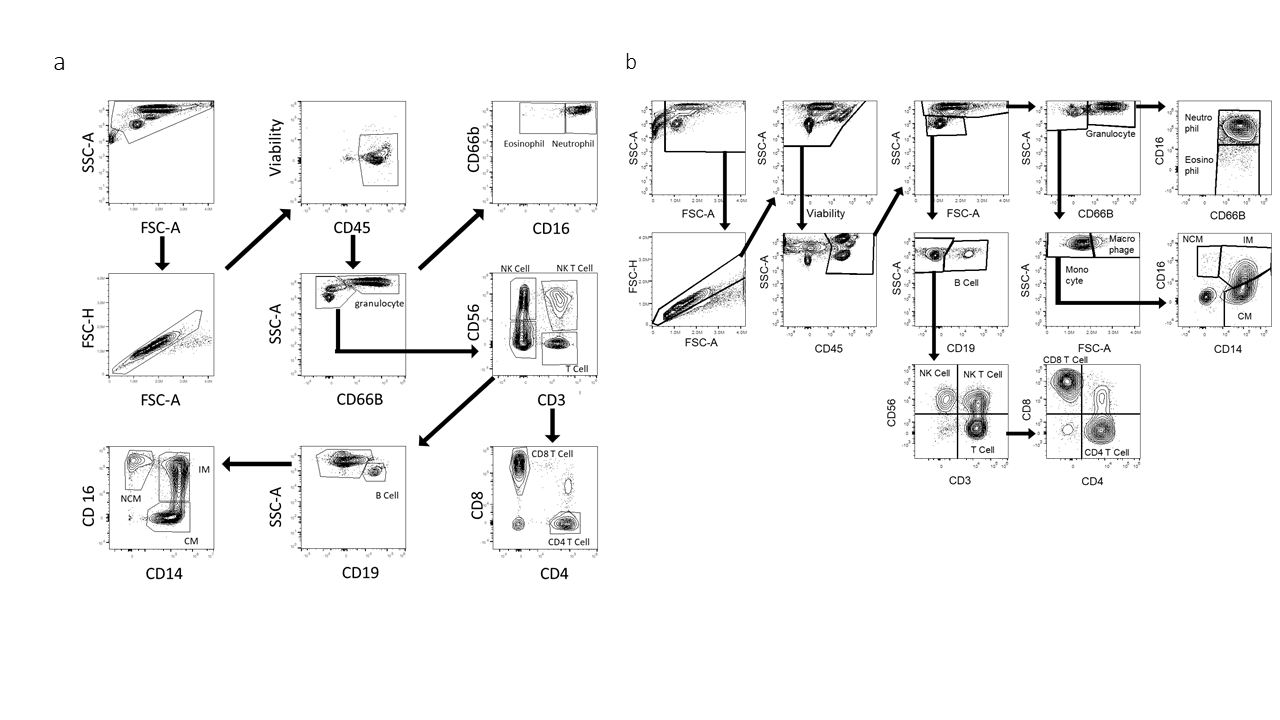

Supplement: Supplementary file 1 [file Image_1.tif]
