# Supplementary material for: The Effect of Normothermic Machine Perfusion on the Immune Profile of Donor Liver
Source: Front Immunol. 2022 Jun 2;13:788935. doi: 10.3389/fimmu.2022.788935 (PMC9201055; doi:10.3389/fimmu.2022.788935)
Supplement: Supplementary file 2 [file Table_1.docx]

**Supplemental Figure Legend**

Supplemental Figure 1. Gating strategy of immune profile. (a) Gating strategy for liver perfusate (Obtained from 0-hour perfusate from liver 10). (b) Gating strategy for liver tissue (Obtained from 0-hour tissue from liver 10). CM- Classical monocyte; NCM- Non-classical monocyte; IM- Intermediate monocyte.

**Supplemental Tables**

Supplemental Table 1. Markers for leukocyte subset characterization using flow cytometry

|  | |
| --- | --- |
| **Leukocyte Characterization** |  |
| Cell Type | Anti-human antibodies |
| Leukocytes | CD45+ |
| T Cells | CD45+, CD3+ |
| CD4 T Cells | CD45+, CD3+, CD4+ |
| CD8 T Cells | CD45+, CD3+, CD8+ |
| B Cells | CD45+, CD19+ |
| NK Cells | CD45+, CD3-, CD56+ |
| NK T Cells | CD45+, CD3+, CD56+ |
| Neutrophils | CD45+, CD66B+, CD16+ |
| Eosinophils | CD45+, CD66B+, CD16- |
| Classical Monocytes | CD45+, CD66B-, CD3-, CD56-, CD19-, CD16-, CD14+ |
| Intermediate Monocytes | CD45+, CD66B-, CD3-, CD56-, CD19-, CD16+, CD14+ |
| Non-classical Monocytes | CD45+, CD66B-, CD3-, CD56-, CD19-, CD16+, CD14- |

Supplemental Table 2. Markers for T cell subset characterization using immunohistochemistry

| **Cells** | Markers |
| --- | --- |
| All cells | DAPI+ |
| T Cells | DAPI+ CD3+ |
| T Regulatory Cells | DAPI+ CD4+FoxP3+ |
